# Glucocorticoid receptor signaling in astrocytes is required for aversive memory formation

**DOI:** 10.1038/s41398-018-0300-x

**Published:** 2018-11-28

**Authors:** Magdalena Tertil, Urszula Skupio, Justyna Barut, Valentyna Dubovyk, Agnieszka Wawrzczak-Bargiela, Zbigniew Soltys, Slawomir Golda, Lucja Kudla, Lucja Wiktorowska, Klaudia Szklarczyk, Michal Korostynski, Ryszard Przewlocki, Michal Slezak

**Affiliations:** 1 Department of Molecular Neuropharmacology, Institute of Pharmacology, Polish Academy of Sciences, Cracow, 31-343 Poland; 2 Team Brain Microcircuits in Psychiatric Diseases, BioMed X Innovation Center, Heidelberg, 69120 Germany; 3 Department of Neuroanatomy, Institute of Zoology and Biomedical Research, Jagiellonian University, Cracow, 30-387 Poland

## Abstract

Stress elicits the release of glucocorticoids (GCs) that regulate energy metabolism and play a role in emotional memory. Astrocytes express glucocorticoid receptors (GR), but their contribution to cognitive effects of GC’s action in the brain is unknown. To address this question, we studied how astrocyte-specific elimination of GR affects animal behavior known to be regulated by stress. Mice with astrocyte-specific ablation of GR presented impaired aversive memory expression in two different paradigms of Pavlovian learning: contextual fear conditioning and conditioned place aversion. These mice also displayed compromised regulation of genes encoding key elements of the glucose metabolism pathway upon GR stimulation. In particular, we identified that the glial, but not the neuronal isoform of a crucial stress-response molecule, *Sgk1*, undergoes GR-dependent regulation in vivo and demonstrated the involvement of SGK1 in regulation of glucose uptake in astrocytes. Together, our results reveal astrocytes as a central element in GC-dependent formation of aversive memory and suggest their relevance for stress-induced alteration of brain glucose metabolism. Consequently, astrocytes should be considered as a cellular target of therapies of stress-induced brain diseases.

## Introduction

Stressful events leave emotional memory traces that enable adaptation of the animal to novel conditions^1,2^. Emotional memories persist longer than memories of neutral events and rely on the interaction between several brain regions, including hippocampus and amygdala^3^. In extreme cases, the exposure to stress may turn into disease, for example post-traumatic stress disorder (PTSD) or depression^1–3^.

Cognitive effects of stress are largely mediated by glucocorticoid hormones (GCs) whose release is controlled via the hypothalamus−pituitary−adrenal (HPA) axis^4,5^. GCs exert their action through their glucocorticoid receptors (GR)^6–9^, which are uniformly distributed across cell types and regions of the central nervous system (CNS) and which regulate gene transcription^10,11^. Genetic variants of GR and GR-dependent genes have been linked to susceptibility to stress-related diseases and they have been shown to predict the outcome of antidepressant treatment^12–14^. Detailed understanding of how GR contributes to stress-induced brain dysfunction may hence lead to more successful therapeutic strategies for stress-induced disorders.

The key question is which cells participate in GR-dependent formation of stress-induced memories. Cell-specific GR elimination revealed a crucial role of neurons in the hippocampus, amygdala, and other brain areas^15–18^. The role of astrocytes, which represent a major cell type in the brain, has not been studied, despite the relevance of GR signaling in astrocytes for psychiatric conditions has been suggested in a survey of transcriptional changes elicited by psychoactive compounds^19^. Astrocytes express GR and respond to GR stimulation with increased intracellular calcium concentrations and alterations of their transcriptional and metabolic profiles^20–27^. Recent research shows that glial metabolic activity involving glucose uptake, glycogenolysis and lactate release is indispensable for memory formation^28–30^. In our previous work^26^ we showed that GR activation in astrocytes leads to upregulation of two protein kinases known to regulate metabolism, namely the pyruvate dehydrogenase kinase-4 (PDK4) that controls entering of glucose into the citric acid cycle and serum and glucocorticoid-regulated kinase-1 (SGK1), a protein associated with PTSD and depression^31^. Due to its clinical relevance, several studies explored the function of SGK1 in neurons^31,32^, while the role of this protein in glial cells remains poorly recognized, despite demonstrated GR-dependent regulation^26^.

Here, we set out to explore whether GR signaling in astrocytes contributes to emotional memory. We used a transgenic approach to selectively eliminate GR from astrocytes in the adult mouse brain and we evaluated the impact of this genetic manipulation on the behavioral outcome in classical paradigms known to rely on the intact GC signaling: fear conditioning^33^ and conditioned place preference or aversion^34,35^. Furthermore, we explored the molecular basis of GC regulation of astrocyte metabolism as a potential mechanism relevant for observed behavioral effects.

## Materials and methods

### Animals and drug administration

All experiments were approved by II Local Ethics Committee of Institute of Pharmacology. Mice were housed 3–5 per cage in rooms with a controlled temperature of 21 ± 2 °C under a 12/12 h light−dark cycle with free access to food and water. Mice for selective, inducible elimination of GR from astrocytes (GR^astroKO^) were obtained by breeding a Cre-driver line expressing tamoxifen (TAM)-inducible version of Cre recombinase under the promoter of astrocyte-specific gap junction protein, connexin−30 (Tg(Gjb6-CreER^T2^)T53–33Fwp)^36^, with the transgenic line carrying critical exons of gene encoding the glucocorticoid receptor (*Nr3c1*) flanked by loxP sites (Nr3c1^tm2Gsc^ line^8^). Animals were bred on C57Bl/6J background and genotyped as previously described^8,36^. In bigenic mice administration of TAM shall lead to excision of the loxP-flanked genomic elements exclusively in subpopulation of cells expressing CreER^T2^, with the efficiency of recombination depending on the brain region (as the activity of the connexin-30 promoter driving CreER^T2^ varies across the brain) and the availability of TAM. A minimal number of mice to ensure experimental groups (designed based on pilot studies) were put in mating and all male littermates were used in experimental cohorts, randomly assigned to experimental groups. No animals were excluded from the analysis. Experimenters were blinded to the genotype of the animals during experiments and when assessing the outcome.

### Tamoxifen and dexamethasone (DEX) treatment

Experimental male mice of 8–10 weeks were treated once daily for 5 days with TAM (Sigma, 100 μl of 20 mg/ml in sunflower oil). Behavioral experiments were initiated at least 3 weeks after the TAM injection to ensure efficient elimination of the GR protein^37^. For biochemical tests animals were injected i.p. with 4 mg/kg DEX (Dexaven, JELFA S.A.) or saline.

### Shock application and test of conditioned fear

The fear conditioning procedure was performed as previously described^38^. Briefly, the test comprised two parts: training and retrieval. In the contextual fear conditioning paradigm, the training lasted 7 min 10 s and consisted of a 2-min acclimation to an automated shock chamber (Ugo Basile, Comerio, Italy), followed by the application of five foot shocks (1 mA, 2 s each). The 1 mA current was selected based on a pilot study, in which WT mice displayed freezing behavior for approx. 50% of time upon exposure to the context 24 h after the training. Shocks were separated with 1 min intervals, during which freezing behavior (defined as immobility, except for respiratory movements) was recorded with charged coupled device camera (The Imaging Source, Germany). In cue fear conditioning paradigm, the shock was applied during the last 2 s of 20-s tone (60 dB) presented to mice five times with a 1-min interval. After the training animals were kept separately until all mice from the homecage have completed training. For contextual fear conditioning, at 1, 3, 5, or 7 days after training, mice were placed in the same chamber as during the training. Freezing behavior was measured for 3 min in each retrieval session. External conditions (odor, lighting, and time of day) remained unaltered throughout the duration of the tests. For cue fear conditioning, at the same timepoints the animals were moved into a different context (different cage floor and walls, different scent) and were presented with the same tone as during training sessions, and the expression of cue-induced fear memory was measured as freezing during 3 min of the tone presentation. The freezing data were analyzed using ANY-maze software (Stoelting, Wood Dale, IL, USA).

### Nociception

Tail flick was performed using analgesia meter (Ugo Basile, Italy). The light beam was applied to animals’ tail and the latency to tail withdrawal or shaking was recorded. To avoid tissue damage, a cut-off latency was set at 9 s. Hot plate test was conducted using Hot Plate Analgesia Meter (COTM, Bialystok, Poland). The mice were placed on the plate heated to 52.5 °C and the latency to the first reaction (rapid reflexive retraction of the paw or licking/biting of the paw) was measured. To avoid tissue damage, a cut-off latency was set at 30 s.

### Open field

Mice were placed in an open field arena (40 × 40 cm) equipped with photocells (Med Associates, St. Albans, VT, USA). Horizontal activity was measured in 30-min sessions.

### Light/dark box test

Each mouse was placed in the middle of the compartment of the two-part apparatus (20 × 20 × 14 cm each) made of black Plexiglas, lit by a dim light (50 lux). The other compartment was made of white Plexiglas and was illuminated with a lamp (300 lux). The 5-min trials were video-recorded and analyzed using ANY-maze software (Stoelting Co., Wood Dale, IL, USA). We measured the number of transitions between compartments, the latency to enter the illuminated compartment, and the total time spent therein.

### Novel object recognition test

The objects to be discriminated were a plastic toy (2–2.5 cm diameter) and a plastic dice (2 cm). Animals were habituated to the open field for 30 min, during locomotor activity test. The next day, they were individually placed for 10 min (acquisition) in the open field in the presence of object A placed in one of the two presentation positions (in the corner, 5 cm from side walls). The exploration time of object A (i.e. when the animal’s snout was directed toward the object at a distance < 1 cm) was manually recorded. Twenty-four hours later the animals were tested in the 10-min retention trial. A recognition index was defined as [*t*B/(*t*A + *t*B)] × 100, where *t*B/*t*A indicate the times the mouse spent exploring the objects B/A, respectively. The type of objects and their positions of presentation during the acquisition and retention phases were counterbalanced across animals.

### Saccharin preference test

Mice were individually housed for 24 h and given a free choice between two bottles containing tap water or 0.1% saccharin solution. To avoid the possible effects of side preference in drinking behavior, the position of the bottles was changed after 12 h. The preference for saccharin was calculated as a percentage of saccharin solution compared to the total amount of liquid consumed.

### Tail suspension test

Mice were suspended by the tail with adhesive tape from an aluminum bar that was set at a height of 30 cm from the ground. The total duration of immobility was calculated over 6 min.

### Conditioned place preference/aversion (CPP/CPA)

On day 1 (preconditioning) mice were placed in the conditioning boxes for 20 min and allowed to freely explore the apparatus. On days 2–4 (conditioning), mice were treated with alternating injections of saline in the morning sessions and morphine (5 mg/kg) or naloxone (10 mg/kg) during afternoon sessions. Immediately after the injection mice were confined to one of the two compartments of the two-chamber box, for 40 min. On day 5 (drug-free test) the mice were allowed to explore the whole apparatus without any treatment and the time spent in the drug-paired compartment was measured.

### Immunofluorescence

Animals were perfused with 4% paraformaldehyde (PFA) buffered with phosphate buffered saline (PBS), brains were postfixed in 4% PFA and stored in PBS. Serial 40-μm-thick coronal sections from CTRL and GR^astroKO^ mice obtained with vibratome were processed in parallel. Free-floating sections were rinsed in PBS, incubated with 10% normal goat serum in PBS with 0.2% Triton X−100 (PBS-Tx) for 90 min at room temperature (RT) and incubated overnight at RT with antibodies: polyclonal anti-S100β (1:250, 287004, Synaptic Systems) or a monoclonal anti-NeuN (1:200, MAB377, Merck Millipore) and a monoclonal anti-GR (1:100, 12041 S, Cell Signalling) diluted in PBS-Tx containing 1% normal serum. The next day, sections were washed in PBS and incubated for 90 min at RT with fluorophore-conjugated secondary antibodies (A11073 and A11036, ThermoFisher), diluted 1:250 in PBS-Tx containing 1% normal serum. Afterwards, sections were washed, incubated for 10 min at RT with Hoechst (1:2000 dilution in PBS, H3570, ThermoFisher), washed and embedded in ProLong Diamond Antifade Mountant (P36965, ThermoFisher). Imaging was performed with a fluorescent confocal microscope (Nikon A1R). The contrast and brightness were altered for illustration purposes in FIJI^39^. Co-expression of GR with S100β or NeuN was determined in individual confocal sections of hippocampal CA1 area, prefrontal cortex (PFC) and amygdala using Synchronize Windows and Cell Counter plugins.

### Magnetic beads-based sorting of astrocytes

Hippocampi from two animals were pooled and lysed into single-cell suspension using Neural Tissue Dissociation Kit (T) (Miltenyi Biotec) on GentleMACS Dissociator. Next, samples were depleted of myelinating cells on LS columns (Miltenyi Biotec) using Myelin Removal Beads II (Miltenyi Biotec). Astrocytes were isolated (Suppl. Figure 1) on MS columns using Anti-Glast Microbead Kit (Miltenyi Biotec). Flow-through from the columns was also collected and analyzed in parallel. RNA was isolated using RNAeasy Micro Kit (Qiagen) and reverse-transcribed into cDNA using Superscript III Reverse Transcriptase (Invitrogen).

### Gene expression analysis

Dissected CNS structures were immersed in RNAlater solution (Qiagen) and stored in −70 °C until isolation. Tissues were homogenized in Trizol reagent (Life Technologies) using TissueLyser (Qiagen). Primary cells were harvested in Trizol. Total RNA (500–1000 ng) was reverse-transcribed into cDNA using Omniscript RT Kit (Qiagen). cDNA corresponding to 5–10 ng of total RNA was used for qPCR utilizing TaqMan Universal PCR Master Mix (Applied Biosystems) and commercial TaqMan probes (Applied Biosystems) as well as Assay-On-Demand probes (Applied Biosystems) for different *Sgk1* variants: *Sgk1_002* encoding a neuron-specific isoform^40^, *Sgk1_003* and a canonical isoform *Sgk1_001*, the latter two enriched in glial cells^26^. Assays were run on the iCycler (Bio-Rad). Data were calculated according to ΔCt method, using *Actb* or *Hprt* as reference genes. Fold change of DEX-induced gene expression was calculated and presented as a ratio over SAL-induced expression.

### Protein expression analysis

Cells were scraped in ice-cold PBS and lysed in RIPA buffer. Thirty-50 μg of protein extract was resolved according to standard SDS-PAGE protocol and transferred onto a nitrocellulose membrane (Protran). Detection was accomplished with anti-SGK1 (ab59337, Abcam) and anti-β-actin (A5441, Sigma) antibodies, resolved with respective secondary antibodies (PI-1000 and PI-2000, Vector Laboratories). Chemiluminescent signal was collected using LAS 4000 Luminescence Image Analyzer (Fuji).

### Primary cultures, viral transduction, and drug treatment in vitro

Brain hemispheres were dissected from 5–7-day-old C57Bl/6N mice and dissociated into a single-cell suspension using Neural Tissue Dissociation Kit (T) (Miltenyi Biotec). Cells were isolated by magnetic sorting using Anti-Glast Microbead Kit and MS Columns (Miltenyi Biotec), seeded on polyornithine (Sigma-Aldrich)-coated dishes and cultured in Dulbecco's modified Eagle medium αemented with 10% fetal bovine serum and penicillin/streptomycin under 5% CO_2_ at 37 °C for 7–10 days, when they were re-plated for experiments. After reaching confluence, cells were grown in serum-free medium for 24 h and stimulated with 100 nM dexamethasone (Sigma) for 4 h or 24 h. For lentiviral vector (LV)-mediated gene knockdown, astrocytes were incubated overnight with LV-shRNA particles at multiplicity of infection = 75 in the presence of 4 μg/ml polybrene (Sigma). Experiments started 72 h post-infection. Details of LV production are provided in Supplementary Methods.

### In vitro assays

Metabolic assays were performed using Glucose Uptake Assay Kit, l-Lactate Assay Kit, and Glycogen Assay Kit (all Abcam) according to the vendor’s protocol. Fluorescence was measured using Tecan Infinite m1000 Pro microplate reader.

### Statistical analyses

Normality and homogeneity of variance were examined using Shapiro−Wilk and Levene tests, respectively. Following Student’s *t* test or two-way ANOVA were applied where appropriate. Two-way ANOVA and post-hoc tests were performed using lm, lme, and emmeans functions (R Core Team, 2018). Full statistical analysis is provided in Supplementary Table.

## Results

### Characterization of a transgenic mouse model to study the role of GR signaling in astrocytes

**Fig. 1:**
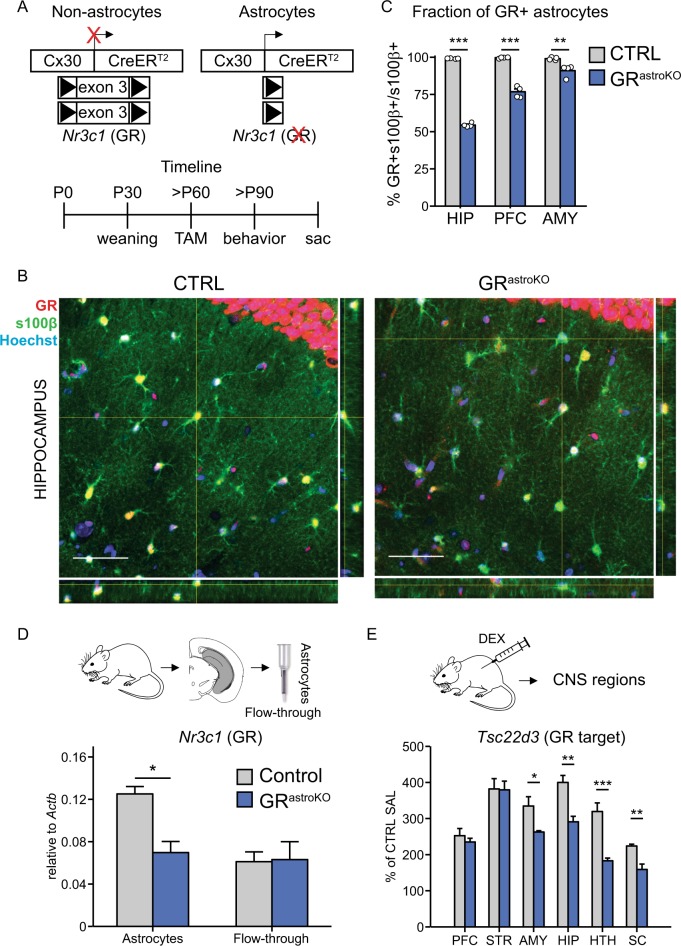
Characterization of a transgenic mouse model for astrocyte-specific elimination of GR. **a** Top: schematic representation of the expression of *Nr3c1* gene (encoding GR) in astrocytes (active Cx30 promoter) or other cells (inactive Cx30 promoter) in bigenic mice carrying Cx30-CreER^T2^ and GR^lox/lox^ alleles. Bottom: experimental timeline. **b** Confocal microphotographs containing single optical slices collected from hippocampal sections from CTRL (left) and GR^astroKO^ (right) mice after immunohistochemical staining against GR (red), an astrocytic marker s100β (green) and nuclear counterstain with Hoechst (blue). Side panels contain orthogonal views of confocal stacks at indicated sites. **c** Bar plot summarizing the fraction of s100β-positive astrocytes displaying GR immunoreactivity in sections of indicated brain regions obtained from CTRL (gray bars, *n* = 1937 astrocytes) and GR^astroKO^ (blue bars, *n* = 1785 astrocytes) mice (*n* = 4 per group). HIP hippocampus, AMY amygdala, PFC prefrontal cortex. **d** The abundance of *Nr3c1* normalized to *Actb* in the purified hippocampal astrocytes and flow-through from the MACS column (see Methods) obtained from CTRL and GR^astroKO^ mice measured with qPCR. **e** The abundance of *Tsc22d3*, normalized sample-wise to *Actb* and normalized to the expression in CTRL mice receiving SAL, examined with qPCR in homogenates of prefrontal cortex (PFC), striatum (STR), amygdala (AMY), hippocampus (HIP), hypothalamus (HTH) and spinal cord (SC) collected from CTRL (*n* = 4) and GR^astroKO^ (*n* = 5) mice 4 h after administration of DEX (4 mg/kg). Mean ± SEM, *p < 0.05, **p < 0.01, ***p < 0.001, *t* test

To test whether astrocyte-specific GR signaling contributes to GR-dependent cognitive effects, we employed a conditional knockout strategy. We selectively eliminated GR from astrocytes by administering tamoxifen (TAM) to offspring originating from breeding the transgenic line carrying TAM-inducible version of Cre recombinase driven by the astrocyte-specific promoter^36^ with the transgenic line carrying critical exons of the *Nr3c1* gene, encoding GR, flanked by loxP sites^8^ (Fig. 1a). We named bigenic, TAM-treated mice GR^astroKO^, while monogenic, TAM-treated mice were used as a control (CTRL). To test for the efficiency of recombination we performed immunohistochemical examination of the expression of GR in astrocytes in sections collected from CTRL and GR^astroKO^ mice (Fig. 1b). This analysis revealed that in several brain regions relevant for emotional memory, namely hippocampus, prefrontal cortex (PFC) and amygdala all astrocytes in CTRL mice expressed GR (Fig. 1c). In contrast, in sections from GR^astroKO^ mice the fraction of astrocytes expressing GR halved in the hippocampus, while in the PFC and amygdala the recombination efficiency was lower (Fig. 1c). To test for the specificity of GR elimination we checked for a fraction of NeuN-positive neurons expressing GR and, as expected, no changes were detected between CTRL and GR^astroKO^ groups (Suppl. Figure 2). The specific elimination of GR was further confirmed by observation that the abundance of the *Nr3c1* transcript approximately halved in astrocytes, but not in the mix of other cell types isolated from hippocampi of GR^astroKO^ mice, as compared to CTRL mice (Fig. 1d). The functional efficiency of our intervention was evaluated by measuring the abundance of a bona fide GR transcriptional target, *Tsc22d3*, in several regions of the CNS collected 4 h after i.p. injection of a GR agonist, dexamethasone (DEX, 4 mg/kg) or saline (Fig. 1e). We found that DEX-induced increase of *Tsc22d3* was diminished in GR^astroKO^ as compared to CTRL mice in homogenates of spinal cord, hypothalamus, hippocampus, and amygdala, but not in the PFC and striatum.

Taken together, these data indicate that our strategy led to the efficient elimination of GR from astrocytes in the hippocampus, with lower efficiency in amygdala and PFC. Simultaneously, we identified astrocytes as a direct and prominent cellular target of GR transcriptional activity in the CNS of adult mice.

### Astrocyte-specific elimination of GR impairs fear memory

**Fig. 2:**
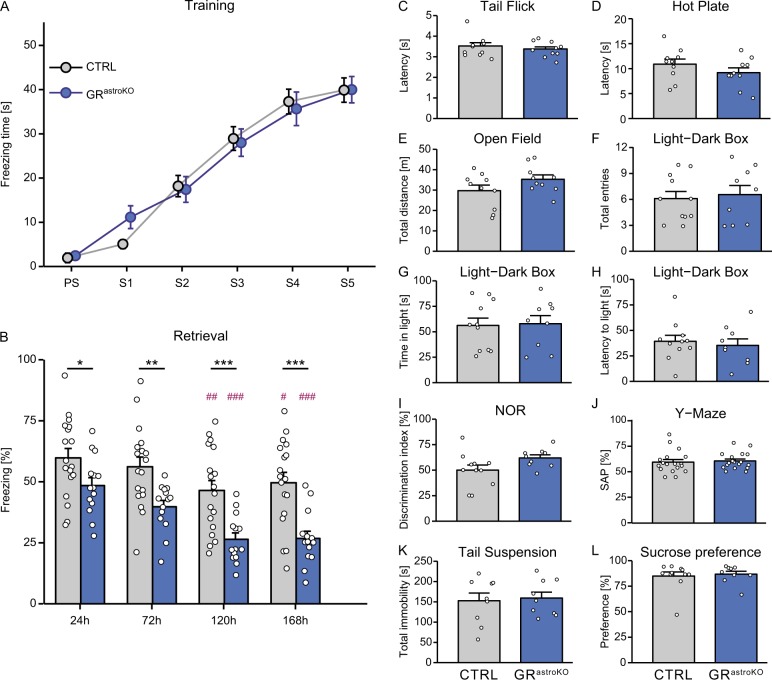
Astrocyte-specific elimination of GR impairs contextual fear memory. **a** Plot summarizing the freezing time of CTRL (gray dots) and GR^astroKO^ mice (blue dots) upon exposure to consecutive electric foot shocks. **b** The analysis of freezing behavior upon exposure to the context during retrieval sessions performed at indicated times after the training. Mean ± SEM, *p < 0.05, **p < 0.01, ***p < 0.001 compared to CTRL for respective timepoints; #*p* < 0.05, ##*p* < 0.01, ###*p* < 0.001, compared to 24 h timepoint in the respective group; two-way repeated measures ANOVA followed by Bonferroni post-hoc test. **c**−**l** The analysis of behavioral parameters in CTRL (gray bars) and GR^astroKO^ (blue bars) mice: nociception assessed by a latency to tail (**c**) or paw (**d**) withdrawal during the tail flick or hot plate test, respectively; locomotor activity assessed by a total distance traveled during the open field test (**e**) or number of transitions between compartments in the light−dark box test (**f**); anxiety assessed by total time spent in (**g**) or latency to enter (**h**) the light compartment of the light−dark box; neutral 24 h memory assessed in the novel object recognition test (**i**); working memory assessed with Y-maze test (**j**); depressive-like behavior assessed by total immobility time in the tail suspension test (**k**); anhedonia assessed in a sucrose preference test (**l**). Mean ± SEM, *t* test

Equipped with the tool, we asked whether astrocytic GR signaling is involved in emotional memory. To answer this question, we exposed CTRL and GR^astroKO^ mice to contextual fear conditioning paradigm, which relies on the intact hippocampal network, where GR ablation from astrocytes in GR^astroKO^ was robust. Mice were habituated to the apparatus for 2 min before receiving five consecutive foot shocks (see Methods). The freezing time measured during the training sessions was similar in both groups (Fig. 2a). Together with the fact that the nociception of GR^astroKO^ mice did not differ from the CTRL mice (Fig. 2c, d), these data confirmed even sensory stimulation in both groups. We measured conditioned fear memory in four retrieval sessions, starting from 24 h and spaced with 48 h intervals. As expected, both groups displayed progressive shortening of freezing time upon repetitive exposure to the fear context. However, two-way repeated measures ANOVA revealed a major effect of genotype (F(1,30) = 19.58, *p* < 0.001) and time (F(3, 90) = 17.31, *p* < 0.001), but not the interaction of the two factors on the freezing behavior. Significant differences between the CTRL and GR^astroKO^ group were detected at every timepoint tested (Fig. 2b). Importantly, in a series of control experiments we excluded the possible effects of the mutation on several behavioral parameters that may confound the interpretation of these results. GR^astroKO^ mice were indistinguishable from the CTRL group with respect to their locomotor behavior (Fig. 2e, f) and anxiety (Fig. 2g, h). We did not detect deficits in 24 h memory of a neutral event (Fig. 2i) nor in working memory (Fig. 2j). Moreover, our genetic manipulation did not elicit depressive-like symptoms, because performance in the tail suspension test (Fig. 2k) and sucrose preference test (Fig. 2l) did not differ between the two groups. The specific role of hippocampal astrocytes in contextual fear memory formation was highlighted by the fact that we did not detect differences between the CTRL and GR^astroKO^ groups in the cue fear conditioning paradigm (Suppl. Figure 3), which relies on intact amygdala (a brain region where GR elimination from astrocytes was minor).

These data reveal that astrocyte-specific GR signaling is involved in fear memory.

### Astrocyte-specific elimination of GR prevents aversive memory formation

**Fig. 3:**
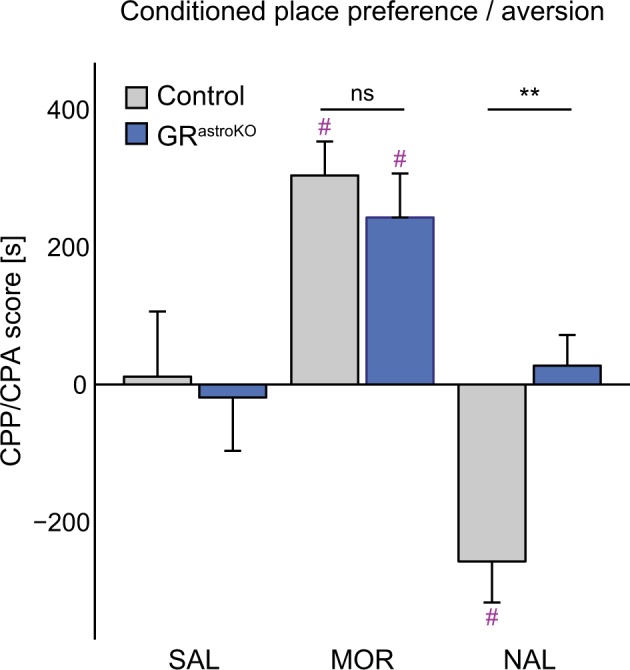
Astrocyte-specific elimination of GR blocks conditioned place aversion. Comparison of the total time spent in the compartment paired with administration of saline (SAL), morphine (MOR) or naloxone (NAL) between CTRL (gray bars) and GR^astroKO^ (blue bars) mice. Mean ± SEM; **p < 0.01 compared to CTRL NAL; #*p*<0.05 compared to respective SAL group; *n* = 13 (CTRL SAL), 12 (CTRL MOR), 19 (CTRL NAL), 14 (GR^astroKO^ SAL), 17 (GR^astroKO^ MOR), 14 (GR^astroKO^ NAL); two-way ANOVA type 3 followed by Bonferroni post-hoc test

Next, we asked whether astrocytic GR signaling is relevant for emotional memories of opposite valence. To examine that, we used two paradigms of Pavlovian conditioning where animals learn to associate the exposure to an appetitive or aversive drug with a specific compartment of the two-chamber box. CTRL and GR^astroKO^ mice could freely explore the two-compartment box for 20 min on day 1 of the procedure. On days 2–4 they received i.p. administration of saline, morphine (MOR) or naloxone (NAL) and immediately after the injection they were placed for 40 min in the compartment paired with the drug. On the test day mice were allowed to explore the entire apparatus and we measured the time that they spent in the drug-associated compartment. Two-way ANOVA (type 3) revealed the significant effect of the treatment (F(2, 83) = 17.76, *p* < 0.001) and genotype × treatment interaction (F(2,83) = 4.21, *p* < 0.05), but not of the genotype on the time that animals spent in a drug-paired compartment. Detailed analysis revealed that both groups developed conditioned place preference (CPP) to morphine to a similar degree (Fig. 3). In contrast, conditioned place aversion (CPA) to naloxone has developed only in the group of CTRL mice, but not in GR^astroKO^ mice (Fig. 3).

Together with the results of fear conditioning, these data indicate the crucial role of astrocyte-specific GR signaling in aversive memory.

### Glucocorticoid signaling regulates metabolic status of astrocytes

**Fig. 4:**
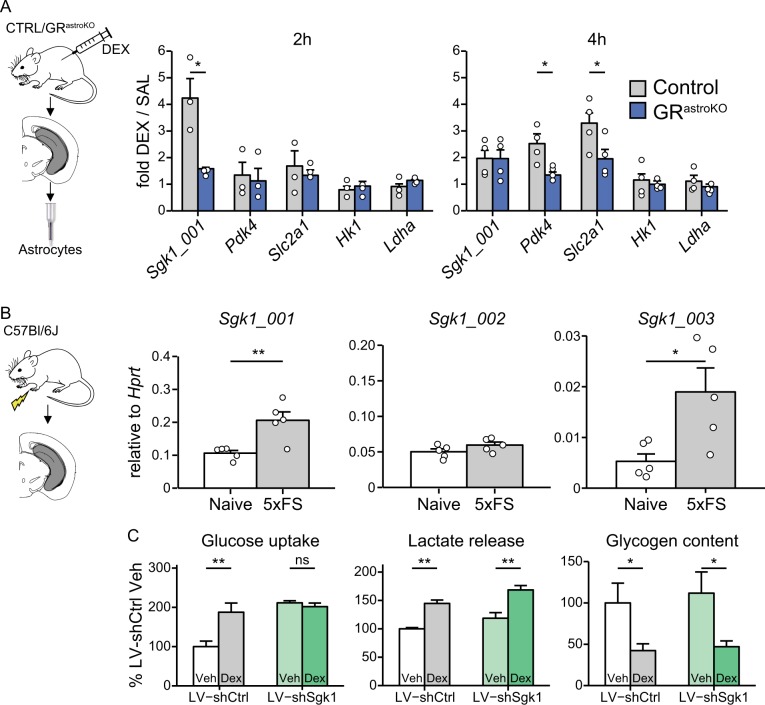
Glucocorticoid signaling regulates metabolic status of astrocytes. **a** Abundance of indicated transcripts in purified astrocytes obtained from hippocampi collected from CTRL (gray bars) or GR^astroKO^ (blue bars) mice 2 h (left) or 4 h (right) after i.p. administration of DEX (4 mg/kg) (mean ± SEM, *p < 0.05, compared to respective SAL group; *n* = 3 for 2 h and *n* = 4 for 4 h timepoint; *t* test). **b** Abundance of the *Sgk1_001*, *Sgk1_002* and *Sgk1_003* isoforms in homogenates of hippocampi obtained from naïve mice or 2 h after the exposure to quintuple foot shock (5xFS) (mean ± SEM, *p<0.05, **p<0.01, t-test). **c** Analysis of the effect of SGK1 knockdown on basal and DEX-induced alterations of glucose uptake, lactate release, and glycogen content in primary mouse astrocytes transduced either with LV-shCtrl or LV-shSgk1 and exposed for 24 h to vehicle or DEX (100 nM), normalized to LV-shCtrl Veh (mean ± SEM, *p<0.05, **p<0.01, *n* = 3 for each group, except in glycogen content (*n* = 4), two-way ANOVA with Bonferroni post-hoc test)

Since astrocytes are known to provide metabolic support for neurons in memory formation and glucocorticoids are powerful modulators of metabolic processes, we next explored how GCs affect metabolic status of astrocytes in vivo. We treated CTRL and GR^astroKO^ mice with DEX, sacrificed animals after 2 or 4 h, isolated hippocampal astrocytes and measured the transcriptional profile of several metabolically relevant genes. We observed the induction of the canonical isoform of *Sgk1 (Sgk1_001)*, followed by the activation of *Pdk4* and a glucose transporter 1 (*Slc2a1*) after 4 h of DEX treatment. All GR-induced effects were abolished by astrocyte-specific ablation of GR (Fig. 4a). We also confirmed that the acute stress, namely exposure to quintuple foot shock, elicited in the hippocampus an increase in the expression of two isoforms of *Sgk1* known to be enriched in glial cells, namely *Sgk1-001* and *Sgk1-003*, but not of the neuron-specific isoform *Sgk1_002* (Fig. 4b). Since SGK1 is a known GR-dependent kinase regulating cellular metabolism, we went on to investigate which processes rely on its intact expression in astrocytes. We found that knockdown of *Sgk1* in primary astrocytes (Suppl. Figure 4) affected glucose uptake but did not alter basal nor DEX-induced lactate release or glycogen content (Fig. 4c). Hence, we identified SGK1 as a regulator of glucose uptake in astrocytes.

These data uncover tight regulation of astroglial metabolic status by GCs.

## Discussion

Stress-induced GCs mediate its adaptive roles, including the emotional memory^1,2^. Decades of research revealed a variety of neuronal and synaptic actions of GCs, mediating its cognitive effects in the brain^41^. We show that non-neuronal cell type, astrocytes, is also an important locus of this action. Our results argue that astrocytes are a direct and prominent locus of GR transcriptional activity in the nervous system. Our new genetic model enables further investigation of the effects of acquired loss of GR in astrocytes and examination of astrocytic pathways relevant for the observed behavioral effects.

Our data recapitulate the inhibition of conditioned fear memory resulting from lesions of hippocampus^42^, as well as pharmacological blockade of GR activation in the hippocampus^43^. Likewise, our results mimic the negative effect of adrenalectomy on the development of CPA^35^. Due to objective reasons, above-mentioned studies did not allow to specify a cellular effector of GR signaling in these regions. We conclude that, besides the described involvement of the neuronal compartment^18^, astrocytes are an overlooked cellular site mediating GCs effects on aversive memory. In contrast, we did not observe the effect of the mutation on morphine-induced CPP. One explanation for these data is that GR signaling in neurons mediates appetitive action of abusive compounds, like cocaine^44^, while the glial GR signaling is dispensable for that aspect. An alternative explanation comes from the fact that in our model GR was not eliminated from several brain regions implicated in appetitive memory, like the striatum. Therefore, we cannot rule out that the role of astrocytic GR in appetitive memory could not be detected in this study due to the properties of the transgenic model^36^.

The stage of the aversive memory affected in the study remains speculative, because in our model astrocytes lack GR before the first exposure to the stressor. Our results speak against an impaired acquisition, since we observed identical increase of freezing response to consecutive shock exposures in the training phase of the fear conditioning and no deficits in working memory in GR^astroKO^ mice. More probable is the impact on consolidation or reconsolidation, occurring several hours after exposure to stress^45^. This notion is supported by the fact that most of GR-dependent astrocytic transcripts are characterized by relatively long mRNA half-lives^19^, corresponding to experience-dependent synaptic rearrangements. Astrocytes have long been considered to contribute to experience-dependent synaptic plasticity by providing metabolic support^46^, and secreting neurotrophic and synaptogenic factors, as well as gliotransmitters^47,48^. Several of potent synaptic effectors are known to undergo GR regulation, including ATP released from astrocytes (through activation of SGK1^49^), glial glutamate transporters^25,50^ or connexin-30^21^, which shapes synaptic transmission relevant for hippocampus-dependent contextual memory^51^. It is also tempting to speculate that astrocytes are the site of convergence of noradrenergic^52^ and GCs crosstalk, required for fear memory consolidation^53^.

Our discovery of the specific function of SGK1 in astrocytes may be of clinical significance. The levels of SGK1 are decreased in patients suffering from PTSD or depression^31,32^, which prompted investigation of the role of this protein in reorganization of neuronal connectivity. However, SGK1 isoforms acquire cell type-specific expression profile^40,54^ with the GR-responsive element present upstream of glial, but not neuronal isoforms^26^. Consequently, our observation of GR-dependent upregulation of glial, but not neuronal isoform in vivo matches previous data where GC-dependent upregulation of *Sgk1* transcript and protein was demonstrated in astrocytes, microglia and oligodendrocytes, but not in neurons^26,49,55,56^. These data highlight the need of investigating the function of stress-induced proteins in the context of the relevant cellular locus.

Our data support the notion that GR stimulation induces expression of *Sgk1* which regulates glucose uptake. We hypothesize that this control is exerted through a mechanism involving regulation of the glucose transporter expression (as suggested by our data, Fig. 4a) and/or distribution (as shown previously in kidney cells^57^). This effect may be engaged in GC-induced alteration of glucose consumption^58^, with further implications for stress-induced memory formation^59^. Our data indicate that ablation of GR in astrocytes disrupts this signaling pathway and may explain the negative impact of the mutation on the aversive memory.

In summary, we discovered that GR signaling in astrocytes is an important part of central effects of stress exerted by GCs. Our data open new pathways of search for more efficient therapies of stress-related disorders, focused on astrocytes as the cellular target.

After completing the manuscript we found that the same transgenic mouse model was used in the study^60^. These authors investigated the role of glucocorticoid receptor signaling in astrocytes in a mouse model of armacologically-induced dopaminergic neurons degeneration.

## Electronic supplementary material

Supplementary Figures

Suppl. Table

Suppl. Methods
