# Supplementary material for: Glucocorticoid receptor signaling in astrocytes is required for aversive memory formation
Source: Transl Psychiatry. 2018 Nov 28;8:255. doi: 10.1038/s41398-018-0300-x (PMC6261947; doi:10.1038/s41398-018-0300-x)
Supplement: Supplementary file 1 — Supplementary Figures [file 41398_2018_300_MOESM1_ESM.pdf]

Supplementary Figure 1

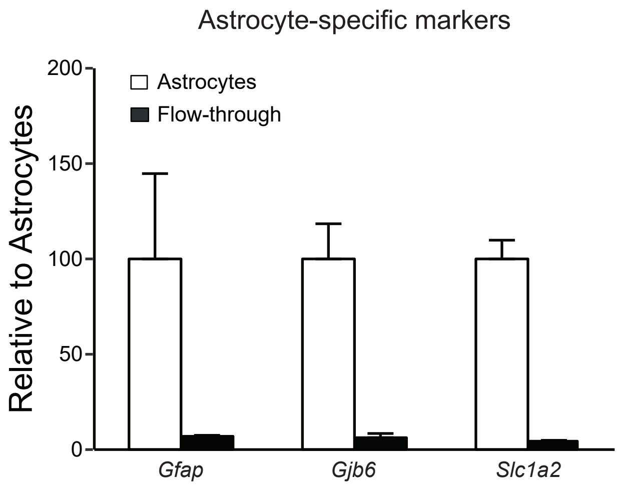

Supplementary Figure 1. Purity of MACS-isolated astrocytes.

Bar plot (mean ± SEM) summarizing the abundance of astrocyte-specific transcripts *Gfap*, *Slc1a2* and *Gjb6* in MACS-sorted population of astrocytes and flow-through from the MACS column, analyzed with qPCR. Data were normalized sample-wise to *Actb*.

Supplementary Figure 2

A.

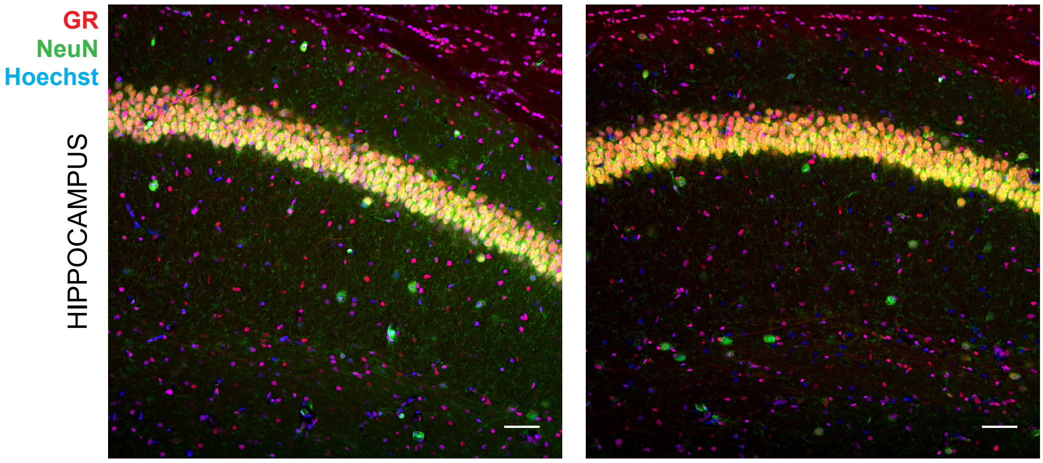

B.

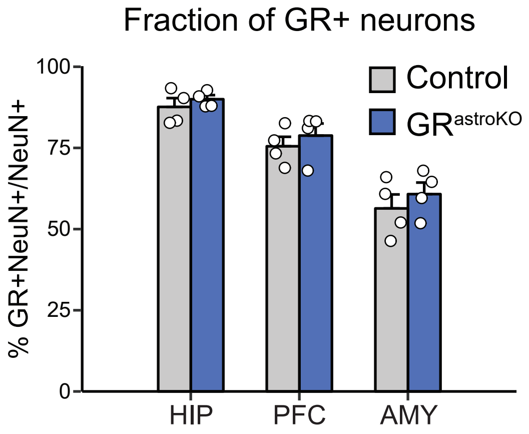

Supplementary Figure 2. No recombination in neurons in GR^{astroKO} mice.

(A) Confocal microphotographs containing single optical slices collected from hippocampal sections from CTRL (left) and GR^{astroKO} (right) mice after immunohistochemical staining against GR (red), a neuronal marker NeuN (green) and nuclear counterstain with Hoechst (blue). (B) Bar plot (mean ± SEM) summarizing the fraction of NeuN-positive cells displaying GR immunoreactivity in sections of indicated brain regions obtained from CTRL (n=4) and GR^{astroKO} (n=4) mice.

Supplementary Figure 3

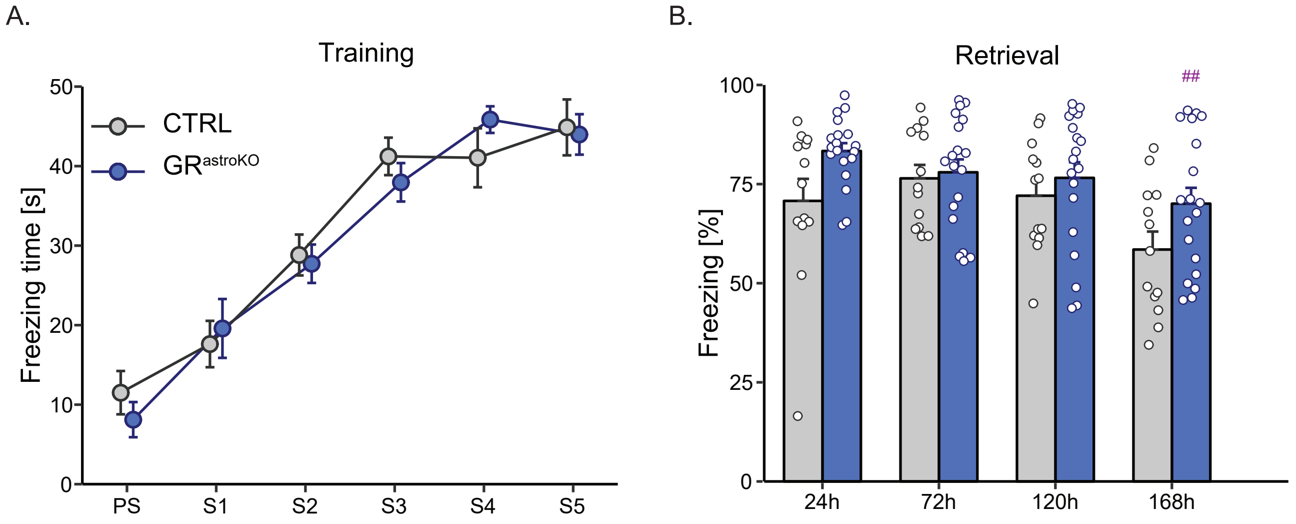

Supplementary Figure 3. Astrocyte-specific elimination of GR does not affect fear memory in cue fear conditioning.

(A) Plot summarizing the freezing time of CTRL (grey dots) and $GR^{astroKO}$ (blue dots) upon exposure to consecutive electric foot shocks preceded by a brief exposure to a tone. (B) The analysis of freezing behavior upon exposure to the cue (tone) during retrieval session performed at indicated times after training. Mean ± SEM, ## $p<0.01$ compared to 24h time point in respective group; two-way repeated measures ANOVA followed by Bonferroni post-hoc test.

Supplementary Figure 4

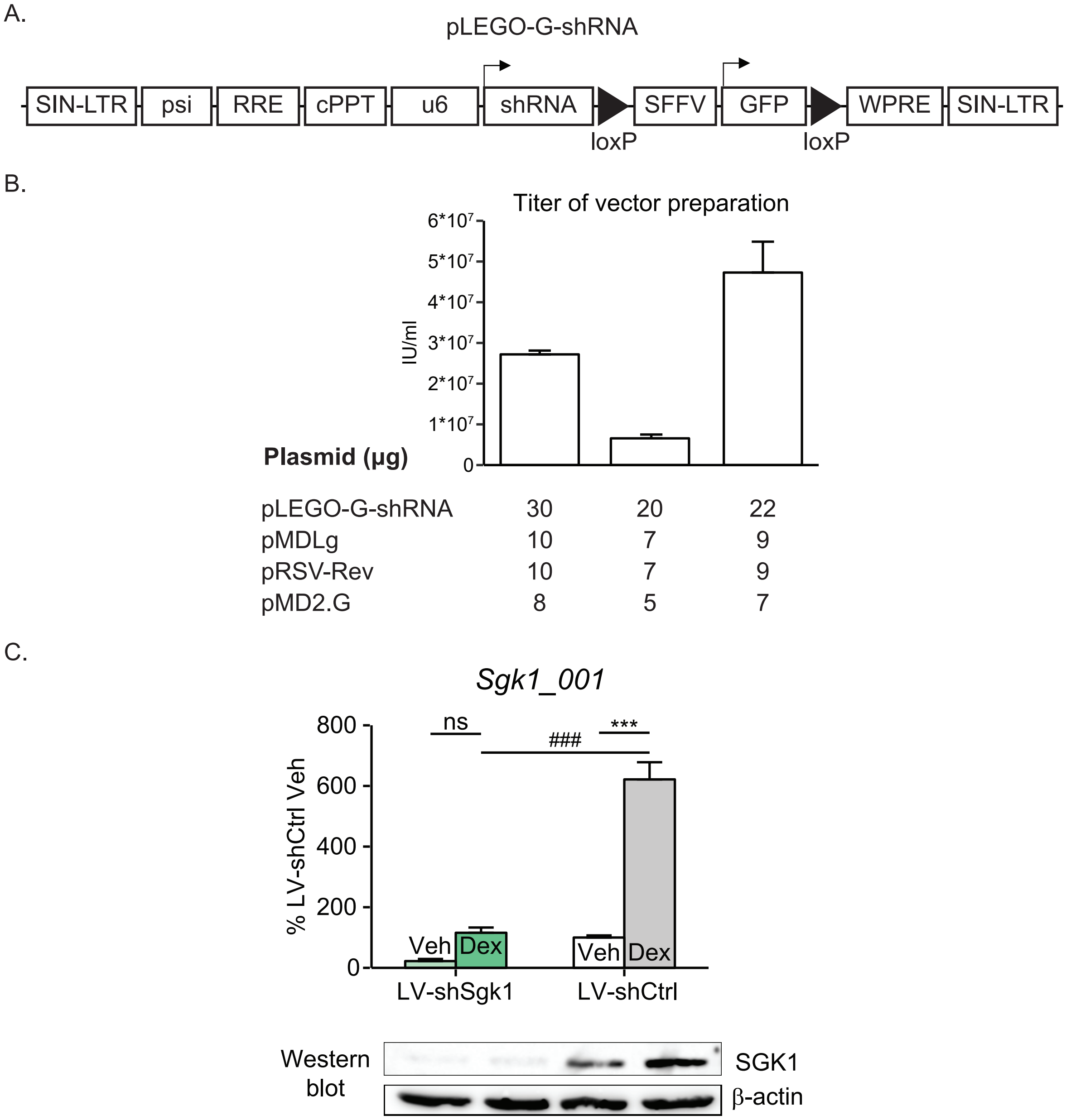

Supplementary Figure 4. Validation of the knock-down of *Sgk*1 in primary astrocytes.

(A) Scheme of the pLEGO-G-shRNA plasmid used for lentiviral vector production. Abbreviations: SIN-LTR, self-inactivating long-terminal repeat; psi, LV packaging signal; RRE, rev-responsive element; cPPT, central poly-purine tract; u6, murine u6 promoter; GFP, green fluorescent protein; SFFV, spleen focus-forming virus U3 promoter; WPRE, Woodchuck hepatitis virus posttranscriptional regulatory element. (B) Bar plot summarizing viral titer collected from supernatant of HEK293 cells co-transfected with the indicated amount of helper plasmids. Abbreviations: pMDLg, plasmid containing the HIV-1 GAG/POL; pRSV-Rev, lentivirus packaging plasmid; pMD2.G, lentivirus envelope plasmid. (C) Top: qPCR detection of the *Sgk1_001* canonical isoform in primary astrocytes transduced either with a lentiviral vector containing shRNA against SGK1 (LV-shSgk1) or its scrambled version (LV-shCtrl) and exposed to vehicle (Veh) or Dex (100 nM, 4h). Data were normalized sample-wise to *Hprt*. Mean ± SEM, *** $p<0.001$ compared to LV-shCTRL Veh; ### $p<0.001$ compared to LV-shCtrl Dex; n=3 for each group; two-way ANOVA followed by Bonferroni post-hoc test. Bottom: microphotograph of Western blots performed with antibodies against SGK1 or β-actin on cell lysates obtained from primary astrocyte cultures treated as in the top panel.
