# Supplementary material for: Glucocorticoid receptor signaling in astrocytes is required for aversive memory formation
Source: Transl Psychiatry. 2018 Nov 28;8:255. doi: 10.1038/s41398-018-0300-x (PMC6261947; doi:10.1038/s41398-018-0300-x)
Supplement: Supplementary file 2 — Suppl. Table [file 41398_2018_300_MOESM2_ESM.pdf]

Summary of statistical analysis

Fig 1C								
GR+S100β+/S100β+	CTRL			GRastroKO				
	Mean	SD	n	Mean	SD	n	p-value	Test
HIP	99.23	0.28	4	54.43	1.4	4	p<0.001	t-test
PFC	99.72	0.56	4	76.8	3.8	4	0.001	
AMY	99.06	0.78	4	90.1	3.58	4	0.006	

Fig 1D								
Nr3c1/Actb	CTRL			GRastroKO				
	Mean	SD	n	Mean	SD	n	p-value	Test
Astrocytes	0.125	0.012	3	0.069	0.018	3	0.011	t-test
Flow-through	0.061	0.016	3	0.063	0.029	3	ns	

Fig 1E								
Tsc22d3/Actb % CTRL Sal	CTRL			GRastroKO				
	Mean	SD	n	Mean	SD	n	p-value	Test
PFC	252.6	39.8	4	235.1	22.9	5	ns	t-test
STR	381.9	57.0	4	379.5	53.6	5	ns	
AMY	334.8	51.4	4	262.6	8.3	5	0.016	
HIP	400.1	38.6	4	290.9	34.3	5	0.003	
HTH	319.5	47.3	4	183.0	17.0	5	<0.001	
SC	224.0	10.1	4	159.0	33.5	5	0.008	

Fig 2B								
Freezing [%]	CTRL			GR astroKO				
	Mean	SD	n	Mean	SD	n	p-value	Test
FC retrieval: 24h	60.2	16.7	18	48.5	12	14	0.030	Two-way repeated measures ANOVA/ Bonferroni post-hoc test
FC retrieval: 72h	54.7	19.5	18	39.8	69.4	14	0.024	
p-value (vs 24h)	ns			ns				
FC retrieval: 120h	46.5	4	18	26.4	2.6	14	<0.001	
p-value (vs 24h)	0.003			<0.001				
FC retrieval: 168h	49.6	4.3	18	26.8	3	14	<0.001	
p-value (vs 24h)	0.039			<0.001				

Fig. 2	CTRL			GR astroKO				
Panel / Parameter	Mean	SD	n	Mean	SD	n	p-value	Test
**C.** Tail flick: latency [s]	3.52	0.51	10	3.38	0.33	10	ns	
**D.** Hot plate: latency [s]	10.88	3.24	10	9.19	3	10	ns	
**E.** Open Field: Total distance [m]	30.29	8.45	10	35.22	7.12	10	ns	
**F.** Light-Dark Box: Total entries	6.09	2.74	11	6.56	3.17	9	ns	

**G.** Light-Dark Box: Time in Light [s]	56.18	23.83	11	57.89	23.72	9	ns	t-test
**H.** Light-Dark Box: Latency to Light [s]	39.36	19.31	11	35.33	18.94	9	ns	
**I.** Novel Object Recognition: Discrimination index [%]	50.04	16.56	11	62.01	9.22	9	ns	
**J.** Y Maze test: Spontaneous Alternation Performance [%]	58.99	10.89	18	60.14	7.9	18	ns	
**K.** Tail suspension test: Total immobility [s]	152.67	56.79	9	159.22	43.85	9	ns	
**L.** Sucrose preference [%]	84.96	13.21	11	86.77	8.75	9	ns	

**Fig 3**								
CPP/CPA score [s]	CTRL			GR astroKO				
	Mean	SD	n	Mean	SD	n	p-value	Test
CPP/CPA: SAL	11.41	341.57	13	-19.11	289.6	14	ns	Two-way ANOVA/ Bonferroni post-hoc test
CPP/CPA: MOR	304.11	171.15	12	242.91	263.65	17	ns	
p-value vs SAL	0.018			0.019				
CPP/CPA: NAL	-257.51	261.03	19	27.24	166.91	14	0.003	
p-value vs SAL	0.015			ns				

**Fig 4A**								
	**2h**							
Fold DEX/SAL normalized to *Hprt*	CTRL			GRastroKO				
	Mean	SD	n	Mean	SD	n	p-value	Test
Sgk1_001	4.23	1.27	3	1.58	0.09	3	0.023	t-test
Pdk4	1.34	0.83	3	1.12	0.81	3	ns	
Slc2a1	1.68	0.99	3	1.33	0.35	3	ns	
Hk1	0.79	0.23	3	0.93	0.31	3	ns	
Ldha	0.91	0.17	3	1.14	0.05	3	ns	
	**4h**							
Fold DEX/SAL normalized to *Hprt*	CTRL			GRastroKO				
	Mean	SD	n	Mean	SD	n	p-value	Test
Sgk1_001	1.97	0.61	4	1.96	0.64	4	ns	t-test
Pdk4	2.52	0.74	4	1.34	0.23	4	0.023	
Slc2a1	3.23	0.76	4	1.95	0.71	4	0.042	
Hk1	1.16	0.46	4	0.99	0.25	4	ns	
Ldha	1.11	0.44	4	0.91	0.19	4	ns	

**Fig 4B**								
relative to *Hprt*	Naive			5xFS				
	Mean	SD	n	Mean	SD	n	p-value	Test
Sgk1_001	0.106	0.018	5	0.206	0.057	5	0.006	

Sgk1_002	0.050	0.009	5	0.060	0.009	5	ns	t-test
Sgk1_003	0.005	0.003	5	0.019	0.011	5	0.025	

**Fig 4C**								
	Veh			Dex				
	Mean	SD	n	Mean	SD	n	p-value	Test
**Glucose uptake**								
LV-shCtrl	100	24.42	3	187.59	40.42	3	0.003	Two-way ANOVA / Bonferroni post-hoc test
LV-shSgk1	211.39	9.37	3	201.76	16.07	3	ns	
p-value	<0.001			ns				
**Lactate release**								
LV-shCtrl	100	3.63	3	144.75	10.26	3	0.002	Two-way ANOVA / Bonferroni post-hoc test
LV-shSgk1	118.59	17.32	3	168.51	13.41	3	0.001	
p-value	ns			0.045				
**Glycogen content**								
LV-shCtrl	100	47.87	4	42.33	23.93	4	0.048	Two-way ANOVA / Bonferroni post-hoc test
LV-shSgk1	111.78	51.24	4	47.11	14.02	4	0.027	
p-value	ns			ns				

**Suppl. Fig 4**								
Sgk1 / Hprt % LV-shCtrl Veh	Veh			Dex				
	Mean	SD	n	Mean	SD	n	p-value	Test
LV-shCtrl	100	12.1	3	621.56	98.12	3	<0.001	Two-way ANOVA / Bonferroni post-hoc test
LV-shSgk1	22.26	12.3	3	115.7	29.96	3	ns	
p-value	ns			<0.001				
