# Supplementary material for: Glucocorticoid receptor signaling in astrocytes is required for aversive memory formation
Source: Transl Psychiatry. 2018 Nov 28;8:255. doi: 10.1038/s41398-018-0300-x (PMC6261947; doi:10.1038/s41398-018-0300-x)
Supplement: Supplementary file 3 — Suppl. Methods [file 41398_2018_300_MOESM3_ESM.docx]

**Development, production and titration of lentiviral vectors used for transduction of astrocytes**.

293T/17 cell line (purchased from ATCC and used at low passages) was cultured in DMEM medium (Life Technologies) supplemented with 10 % fetal bovine serum (FBS, Life Technologies) and penicillin/streptomycin (Life Technologies). Human U6 promoter-driven shRNA expression cassettes (control, Ctrl and targeting *Sgk1*) obtained from The RNAi Consortium (TRC2) were amplified by PCR from pLKO.5 vector, cloned into third generation lentiviral vector plasmid pLeGO-G using *XbaI* and *XhoI* restiction sites and verified by sequencing. For production of the LV, 293T/17 cells seeded on T75 flasks were transfected at approx. 70 % confluence with plasmids: 22 μg of pLeGO-G-shRNA, 9 μg of pMDLg, 9 μg of pRSV-Rev and 7 μg of pMD2.G using linear polyethylenimine (PEI) at 2.58 μg polymer / 1 μg pDNA. Polyplexes were prepared in 150 mM NaCl for 15 min, added dropwise onto the cells and incubated overnight. LV-containing medium was collected 48h post-transfection. For titering of LV, confluent 293T/17 cells were infected with serial dilutions of vector preparations in the presence of 4 μg/mL polybrene (Sigma). 72h post-transduction, eGFP-positive cells were counted under the microscope.
